# An Anomalous Cause of Deep Venous Thrombosis: A Case Report

**DOI:** 10.5811/cpcem.2021.4.51517

**Published:** 2021-06-01

**Authors:** Jana Florian, Huy A. Duong, Jennifer S. Roh

**Affiliations:** * University of California, Irvine, Department of Emergency Medicine, Orange, California; † University of California, Irvine School of Medicine, Irvine, California

**Keywords:** May-Thurner syndrome, deep venous thrombosis, case report

## Abstract

**Introduction:**

Lower extremity deep venous thrombosis (DVT) is a common diagnosis in the emergency department (ED). Deep venous thromboses can be the result of anatomical variation in the vasculature that predisposes the patient to thrombosis. May-Thurner syndrome (MTS) is one such anatomic variant defined by extrinsic compression of the left common iliac vein between the right common iliac artery and lumbar vertebrae.

**Case Report:**

We report such a case of a 39-year-old woman with no risk factors for thromboembolic disease who presented to the ED with extensive unilateral leg swelling and was ultimately diagnosed with MTS.

**Conclusion:**

This diagnosis is an important consideration particularly in patients who are young, female, have scoliosis or spinal abnormalities, or are at low risk for DVT yet who present with extensive lower extremity swelling and are found to have proximal thrombus burden. Often further imaging, anticoagulation, angioplasty, or thrombectomy are indicated to prevent morbidity and post-thrombotic syndrome in these patients.

## INTRODUCTION

Lower extremity deep venous thrombosis (DVT) is frequently diagnosed in the emergency department (ED) setting. Risk factors include malignancy, recent major surgery, trauma, obesity, pregnancy, prolonged immobilization, and hormone therapy. In addition, anatomical abnormalities can also predispose patients to DVT. One such frequently recognized anatomical variant is May-Thurner syndrome (MTS), defined by extrinsic compression of the left common iliac vein between the right common iliac artery and lumbar vertebrae. This compression can lead to venous congestion and, ultimately, left iliofemoral DVT.

The incidence of MTS ranges between 18–49% among patients with left lower extremity DVT.^1^ It is three times more common in women than men, and typically presents in patients between ages 30–40.^2^ Most cases of MTS are asymptomatic and do not require treatment. Symptomatic MTS, however, frequently presents as DVT and requires intervention beyond medical management with anticoagulation. Angioplasty, stenting, and/or thrombectomy prevent complications and minimize morbidity and mortality. Failure to identify MTS as a cause of DVT results in suboptimal treatment and often recurrent thrombosis. May-Thurner syndrome is an essential consideration for the emergency physician in the differential diagnosis of unilateral leg swelling.

## CASE REPORT

A 39-year-old woman with history of alcohol use disorder presented to our ED with two days of atraumatic left leg swelling and pain. She had no risk factors for DVT and no personal or family history of thromboembolic disease or hypercoagulability. Physical exam revealed significant swelling of the left lower extremity with positive Homans’ sign and severe pitting edema extending from the foot to the groin. The extremity was well perfused, hyperemic, with palpable distal pulses. We initially considered uncomplicated DVT, cellulitis, lymphedema, thrombophilic disease, and early phlegmasia cerulea dolens.

Doppler ultrasonography revealed a large occlusive thrombus in the left common femoral vein extending inferiorly to the great saphenous vein. Given the severity of the patient’s symptoms, lack of significant DVT risk factors, and extensive thrombus found on ultrasound, a computed tomography (CT) venogram was obtained to evaluate for underlying structural pathology. Computed tomography showed a fully occlusive filling defect in the left common iliac and femoral veins, extending into the distal inferior vena cava, with evidence of compression of the left iliac vein by the overlying artery, suggestive of May-Thurner syndrome (MTS). A heparin drip was initiated, and the patient was admitted to the hospital for further management.

Given the severity of her symptoms and risk for post-thrombotic syndrome, interventional radiology (IR) was consulted during her admission. The patient elected to proceed with IR-guided thrombectomy, angioplasty, and stenting the following day. The procedure showed a stenotic region of the common iliac vein, confirming a diagnosis of MTS. A 14-millimeter venous stent was placed in this area after pharmacomechanical thrombectomy. She had an uneventful postoperative course, with near-resolution of her symptoms, and was discharged three days later with rivaroxaban and life-long clopidogrel therapy.

## DISCUSSION

Anatomic variants are important but often overlooked risk factors for DVT. May-Thurner syndrome is the most common structural anomaly that predisposes patients to left lower extremity DVT (Figure).

**CPC-EM Capsule**

What do we already know about this clinical entity?

*Lower extremity deep venous thrombosis (DVT) can be caused by anatomic variants of the vasculature*.

What makes this presentation of disease reportable?

*This patient was at low risk for venous thromboembolism yet with significant thrombus burden due to anatomic compression of the iliac vein: May-Thurner syndrome (MTS)*.

What is the major learning point?

*Patients with DVT due to MTS benefit from treatment with a combination of chemical and mechanical intervention, as opposed to sole anticoagulation*.

How might this improve emergency medicine practice?

*Providers should assess for anatomic variants as the etiology of DVT, which requires mechanical intervention to prevent long-term disability*.

The proposed pathogenesis behind symptomatic MTS is the development of a venous spur. The arterial pulsations of the right iliac artery damage the connective tissue in the iliac vein, leading to the development of a DVT.^1^,^3^ Risk factors for MTS include female gender, scoliosis, pregnancy or the postpartum period, and hormone therapy. Importantly, it is also prudent for the emergency physician to have a high index of suspicion for MTS in patients who have *no* risk factors for hypercoagulability and yet present with significant left leg swelling, particularly proximal swelling, and/or recurrent DVT.^4^ The presence of significant symptoms and thrombus burden with a lack of predisposing factors for thromboembolism can be a clue to underlying structural or anatomical variation as the etiology.

In terms of evaluation, ultrasound is the mainstay of initial diagnostic imaging in cases where DVT is suspected. Unfortunately, ultrasound cannot visualize DVT proximal to the groin or external compression of the iliac vein. Thus, further imaging using computed tomography (CT) or magnetic resonance (MR) venography is necessary to diagnose MTS. The use of CT venography has been shown to provide rapid and reliable detection of compression syndromes and is the gold standard for diagnosis.^5^ Overall, certain patient factors and exam findings should raise the clinical suspicion for MTS and prompt further imaging with CT venography in the ED where DVT is suspected or confirmed. These include female gender, scoliosis or spinal abnormalities, extensive left-sided or recurrent DVT, or new diagnosis of DVT in an otherwise low-risk patient.

There are several unique considerations in the management of DVT caused by MTS. Importantly, treatment with anticoagulation alone results in suboptimal outcomes as it does not address the underlying structural pathology of this condition. This can lead to re-thrombosis, recurrent symptoms, iliac vein rupture, and chronic venous stasis. Thus, the first line treatment of MTS is mechanical management with endovascular treatment, namely angioplasty and stenting. This approach reduces the risk for recurrent DVT and long-term negative sequelae such as post-thrombotic syndrome.^6^

Post-thrombotic syndrome is a common complication of DVT and is thought to be caused by venous hypertension from outflow obstruction due to a thrombus. Symptoms are similar to other causes of venous insufficiency, ranging from minor leg swelling to significant leg pain with ulceration.^7^ In one study comparing the use of anticoagulation alone vs stent implantation plus anticoagulation after thrombectomy, 72% of patients had postoperative re-thrombosis as opposed to 13% of patients who underwent stent placement.^6^ Furthermore, there have been a number of other case reports and reviews that advocate for the successful use of endovascular stent placement in long-term treatment of DVT secondary to MTS.^8^,^9^ Overall, angioplasty and stent placement significantly improve quality of life for patients and continue to be the evidence-based method of treatment.

Notably, there is no consensus on the optimal antithrombotic regimen for MTS following venous stenting. Some have advocated for an aggressive approach with both anticoagulation and antiplatelet therapy to prevent re-thrombosis and stent failure.^10^ This was the treatment approach for the patient presented here. Others have suggested sole anticoagulation for at least six months after stent placement. Unfortunately, there is a paucity of literature on the optimal anticoagulation regimen and the exact duration of treatment. Studies report use of a variety of agents, including warfarin, enoxaparin, and direct oral anticoagulants such as rivaroxaban and apixaban.

To date, no randomized controlled trials have been conducted to establish efficacy of one anticoagulation regimen over another. However, most data supports the need for some form of post-procedural anticoagulation.^11^ In addition, studies have shown better outcomes in terms of preventing stent thrombosis with longer duration of anticoagulation therapy. One such systematic review showed that six months of warfarin anticoagulation yielded a 78% 12-month stent patency rate, compared to 89% when warfarin anticoagulation extended beyond six months.^12^ Overall, further investigation is required to determine the optimal antithrombotic regimen for MTS patients after endovascular treatment.

## CONCLUSION

It is essential for emergency physicians to consider anatomic variants such as MTS in the evaluation of patients with unilateral leg swelling. In this case report, we highlight MTS as an important anatomical cause of DVT, especially in young women with left lower extremity swelling and patients without other significant risk factors for thromboembolic disease. Cases are often missed, as the diagnosis of MTS in the ED setting requires a high index of suspicion and CT or MR venography, which is not part of the standard workup for DVT. Prompt recognition of this condition and referral for advanced mechanical or thrombolytic intervention can significantly decrease morbidity and ischemic complications.

## Figures and Tables

**Figure:**
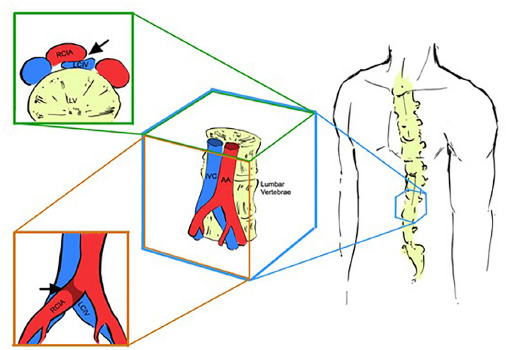
Area of compression (black arrows) in May-Thurner syndrome, as viewed in the coronal (top left) and sagittal planes (bottom left) (medical Illustration design by Dylan Ma). *IVC*, inferior vena cava; *AA*, abdominal aorta; *RCIA*, right common iliac artery; *LCIV*, left common iliac vein.
